# Volatile organic compounds from *Paenibacillus polymyxa* KM2501-1 control *Meloidogyne incognita* by multiple strategies

**DOI:** 10.1038/s41598-017-16631-8

**Published:** 2017-11-24

**Authors:** Wanli Cheng, Jingyan Yang, Qiyu Nie, Dian Huang, Chen Yu, Longyu Zheng, Minmin Cai, Linda S. Thomashow, David M. Weller, Ziniu Yu, Jibin Zhang

**Affiliations:** 1 State Key Laboratory of Agricultural Microbiology and National Engineering Research Center of Microbial Pesticides, College of Life Science and Technology, Huazhong Agricultural University, Wuhan, 430070 Hubei China; 2 United States Department of Agriculture, Agricultural Research Service, Wheat Health, Genetics and Quality Research Unit, Pullman, WA 99164-6430 USA

## Abstract

Plant-parasitic nematodes (PPNs) cause serious crop losses worldwide. In this study, we investigated the nematicidal factors and the modes and mechanisms of action involved in nematode control by *Paenibacillus polymyxa* KM2501-1. Treatment of the second-stage juveniles (J2) juveniles of PPN *Meloidogyne incognita* with the biological control agent KM2501-1 resulted in a mortality of 87.66% *in vitro* and reduced symptoms on tomato by up to 82.61% under greenhouse conditions. We isolated 11 volatile organic compounds (VOCs) from strain KM2501-1, of which 8 had contact nematicidal activity, 6 had fumigant activity, and 5 acted as stable chemotactic agents to *M*. *incognita*. The VOCs provided a comprehensive strategy against PPNs that included “honey-trap”, fumigant, attractant and repellent modes. Furfural acetone and 2-decanol functioned as “honey-traps” attracting *M*. *incognita* and then killing it by contact or fumigation. Two other VOCs, 2-nonanone and 2-decanone, as well as strain KM2501-1 itself, destroyed the integrity of the intestine and pharynx. Collectively our results indicate that VOCs produced by *P*. *polymyxa* KM2501-1 act through diverse mechanisms to control *M*. *incognita*. Moreover, the novel “honey-trap” mode of VOC–nematode interaction revealed in this study extends our understanding of the strategies exploited by nematicidal biocontrol agents.

## Introduction

Plant-parasitic nematodes (PPNs) are among the most economically important pests, causing estimated losses worth more than US $157 billion each year in a broad range of plants and agricultural crops worldwide^1–3^. Root knot nematodes (RKNs; *Meloidogyne* spp.) are the most damaging of the crop PPNs, and are capable of infecting almost all crops^3,4^. *Meloidogyne incognita* is a root knot nematode species with a wide host range and distribution area and is one of the most damaging agricultural nematode species^5^. The use of chemical nematicides remains the primary means of control for PPNs. However, the potential negative environmental impacts have led to a total ban of the most effective nematicides, and the development of resistance has restricted the use of many others. This has created an urgent need for environmentally acceptable and sustainable nematicidal strategies^6^. Biological control promises to be such an option. Application of microorganisms antagonistic to nematodes, or of the compounds produced by these microbes, could provide an additional opportunity for managing the damage caused by RKNs^7^.

Biological control has shown promise as an economically and ecologically friendly approach to reduce nematode damage. To develop biological nematicides, however, further investigation of novel environmental microorganisms is essential^8^ because until now only a few bacteria have been reported to possess nematicidal activity and to show potential for effective control of RKNs^8,9^. Identification of novel biological control agents and the metabolites they produced has become an urgent need for the control of RKNs and is of vital practical and economic significance.

*Paenibacillus polymyxa*, previously described as a species of the genus *Bacillus*, was reclassified in the new genus *Paenibacillus* in 1994^10^. It is a common soil bacterium belonging to plant growth promoting rhizobacteria (PGPR) and produces many beneficial bioactive substances including antibiotics polymyxins, fusaricidins, and antimicrobial proteins that display broad-spectrum antifungal and antibacterial activity^11–13^, as well as phytohormones, that can promote plant growth^14^. Some strains of *P*. *polymyxa* stimulate plant growth via nitrogen, phosphorus and potassium uptake in nutrient deficient soils^15^. While *P*. *polymyxa* is seldomly reported for its nematicidal activity, strain GBR-1 has been reported to suppress RKNs in pot experiments^7^, and two other species of *Paenibacillus* suppressed a disease complex caused by a root-knot nematode and a fusarium wilt fungus^16^. As a sporeformer, *P*. *polymyxa* is an ideal candidate for development as a nematicide because it can easily be formulated for agricultural use.

*P*. *polymyxa* produces antifungal and insecticidal volatile organic compounds (VOCs) as determined by solid phase microextraction-gas chromatography-mass spectrum (SPME-GC-MS)^17,18^. Such compounds can inhibit fungal mycelium growth and the germination of many fungal plant pathogens^19^, and play a vital role in recognition between entomopathogenic nematodes and their hosts^20^. VOCs also are effective against PPNs^18,21^ and recently have been found to exhibit strong nematicidal and fumigant activity against *M*. *incognita* ^21^. This fumigant effect is of great importance because it helps to reach target nematodes that reside far from nematicides in the soil^22^.

That PPNs locate their plant hosts by chemoreception was proposed over 90 years ago^23^, and has since been demonstrated *in vitro* by chemotaxis assays on agar-filled dishes^24–27^. It has been suggested that the use of some compounds like neuropeptides to control nematode behavior might be an efficient way to reduce the infection levels of PPNs^28^. We hypothesize that a similar approach based on VOCS with which PPNs have a stable chemotactic response might also serve as a novel nematicidal mechanism.

We previously isolated *P*. *polymyxa* KM2501-1, a strain with highly nematicidal activity against *M*. *incognita*. In this study, we evaluated the nematicidal activity of *P*. *polymyxa* KM2501-1 against *M*. *incognita in vitro* and in the greenhouse. VOCs produced by the strain were isolated and identified, and their mechanisms of action were explored. The results of these studies indicate that KM2501-1 produces multiple nematicidal VOCs with diverse modes of action, including VOCs that exhibit a novel “honey-trap” mechanism involved in luring and then killing *M*. *incognita*.

## Results

### Nematicidal activity of KM2501-1 *in vitro* and in the greenhouse

**Figure 1:**
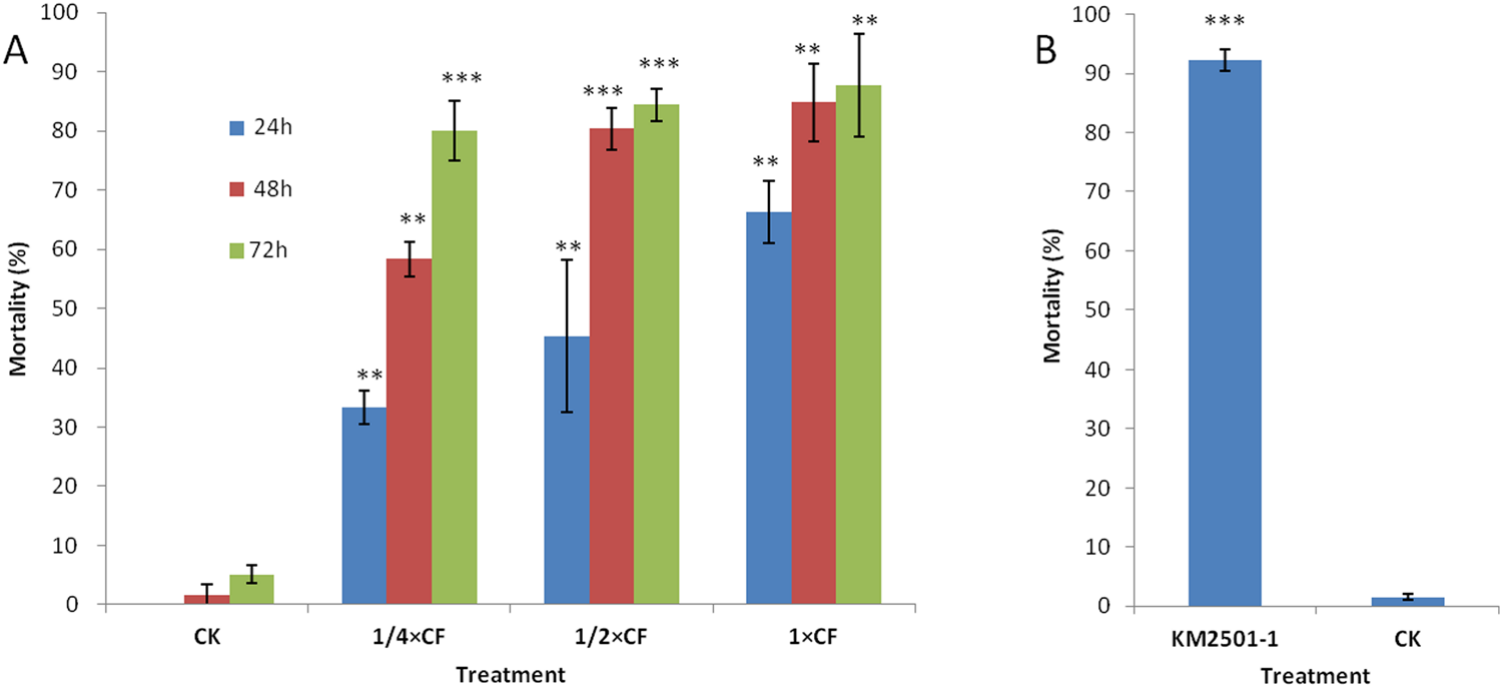
Bioassay of *P*. *polymyxa* KM2501-1 against *M*. *incognita*. (**A**) Nematicidal activity of *P*. *polymyxa* KM2501-1 culture filtrate (CF) at various concentrations and control group (CK) against *M*. *incognita* immersed in treatment wells. (**B**) The nematicidal activity of VOCs of *P*. *polymyxa* KM2501-1 and control group (CK) in a three-compartment Petri dish. The data are shown as the mean ± SD (n ≥ 3). Statistical comparisons between the values of samples and control (CK) were performed using a *t*-test. Significant differences were determined according to a threshold of *P* < *0.05; **P < 0.01, and ***P < 0.001.

Culture filtrates (CF) of KM2501-1 diluted 1:3 (1/4×), 1:1(1/2×) or not diluted (1×) were highly toxic to J2 juveniles of *M*. *incognita*, causing mortality rates of 80.00% to 87.66% at 72 h, whereas the mortality in the control group (CK) was only 5.12% (Fig. 1A and Table S1). When CF of KM2501-1 was tested against *M*. *incognita*, clear dose response relationships and significant mortality of J2 juveniles were evident after 24, 48, or 72 h of exposure. These results indicate that *P*. *polymyxa* KM2501-1 has strong nematicidal activity against *M*. *incognita*, and are consistent with previous reports that some strains of *P*. *polymyxa* are effective against PPNs^7,16,29^. *P*. *polymyxa* KM2501-1 also produced nematicidal VOCs that caused 92.30% mortality of *M*. *incognita*, whereas, the mortality of the control group (CK) was less than 2% in the three-compartmented Petri dish assay (Fig. 1B). The results indicated that *P*. *polymyxa* strain KM2501-1 can produce extracellular nematicidal VOCs.

In the greenhouse, *M*. *incognita* infected and formed numerous large root galls on the control roots of tomatoes, while fewer and smaller galls were observed on roots after treatment with culture filtrate (CF) or bacterial suspension (BS) of *P*. *polymyxa* KM2501-1 (Fig. S1). The root gall index of the control group (CK), the undiluted CF-treated plants, and undiluted BS-treated plants were 4.80, 0.60, and 2.40, respectively in the first set of pot experiment, and were 4.40, 1.00, and 2.40 respectively in the second set of pot experiment (Table 1). The root gall index of all dilutions (1/20x , 1/10x , 1/4x and 1x ) of the BS and CF also were significantly (P < 0.05) lower than that of the control (CK), and the undiluted CF was the most effective.

**Table 1:** Suppression of *Meloydogyne incognita* by *Paenibacillus polymyxa* KM2501-1 in greenhouse pot experiment.

Treatment	Root gall index	
	Experiment 1	Experiment 2
CK	4.80 ± 0.45 a	4.40 ± 0.55 a
1 × CF	0.60 ± 0.55 d	1.00 ± 0.00 d
1/4 × CF	2.60 ± 0.55 c	2.80 ± 0.45 c
1/10 × CF	2.80 ± 0.45 c	2.80 ± 0.45 c
1/20 × CF	3.40 ± 0.55 bc	3.00 ± 0.00 bc
1 × BS	2.40 ± 0.89 c	2.40 ± 0.55 c
1/4 × BS	3.00 ± 0.71 bc	3.30 ± 0.71 bc
1/10 × BS	3.40 ± 0.55 bc	3.40 ± 0.55 bc
1/20 × BS	3.60 ± 0.55 b	3.40 ± 0.55 bc

CK, water was used as control group. CF, culture filtrates of KM2501-1. BS, bacterial suspensions of KM2501-1. The data in experiment 1 and 2 are shown as the mean ± SD (n = 5). Duncan’s multiple range test was employed to test for significant differences between treatments at P < 0.05. Different lowercase letters indicate significant difference between treatments (P < 0.05).

### VOCs from the *P*. *polymyxa* KM2501-1 have contact nematicidal activity

**Figure 2:**
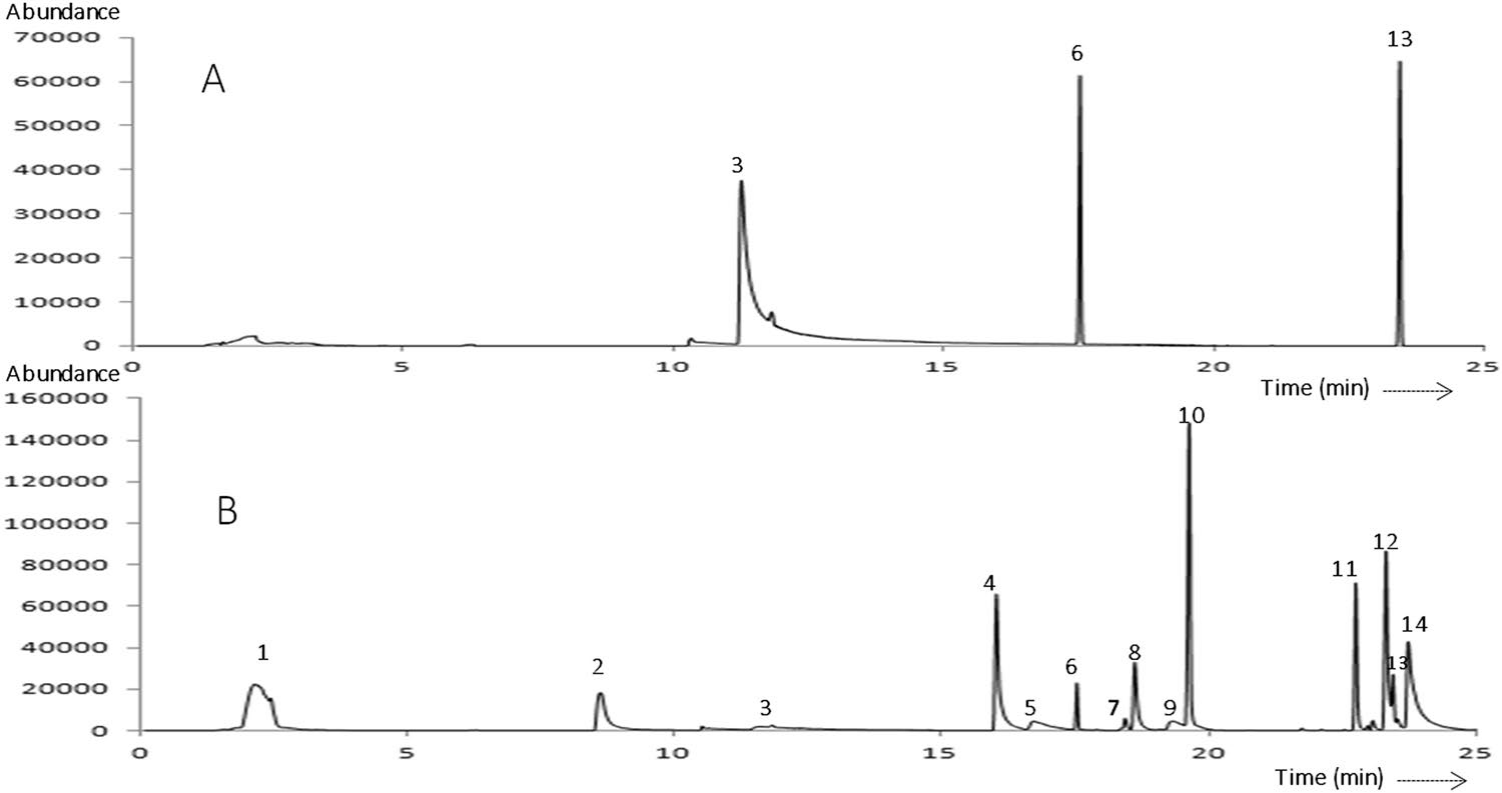
GC-MS chromatograms of (A) KMB, and (B) fermentation broth of KM2501-1. Peaks: (1) acetone, (2) 2-heptanone, (3) benzaldehyde, (4) 2-nonanone, (5) 2-nonanol, (6) cyclopentasiloxane, decamethyl-, (7) 11-dodecen-2-one, (8) 2-decanone, (9) 2-decanol, (10) 4-acetylbenzoic acid, (11) furfural acetone, (12) 2-undecanone, (13) Acetic acid, [bis[(trimethylsilyl)oxy]phosphinyl]-, trimethylsilyl ester, (14) 2-undecanol.

**Figure 3:**
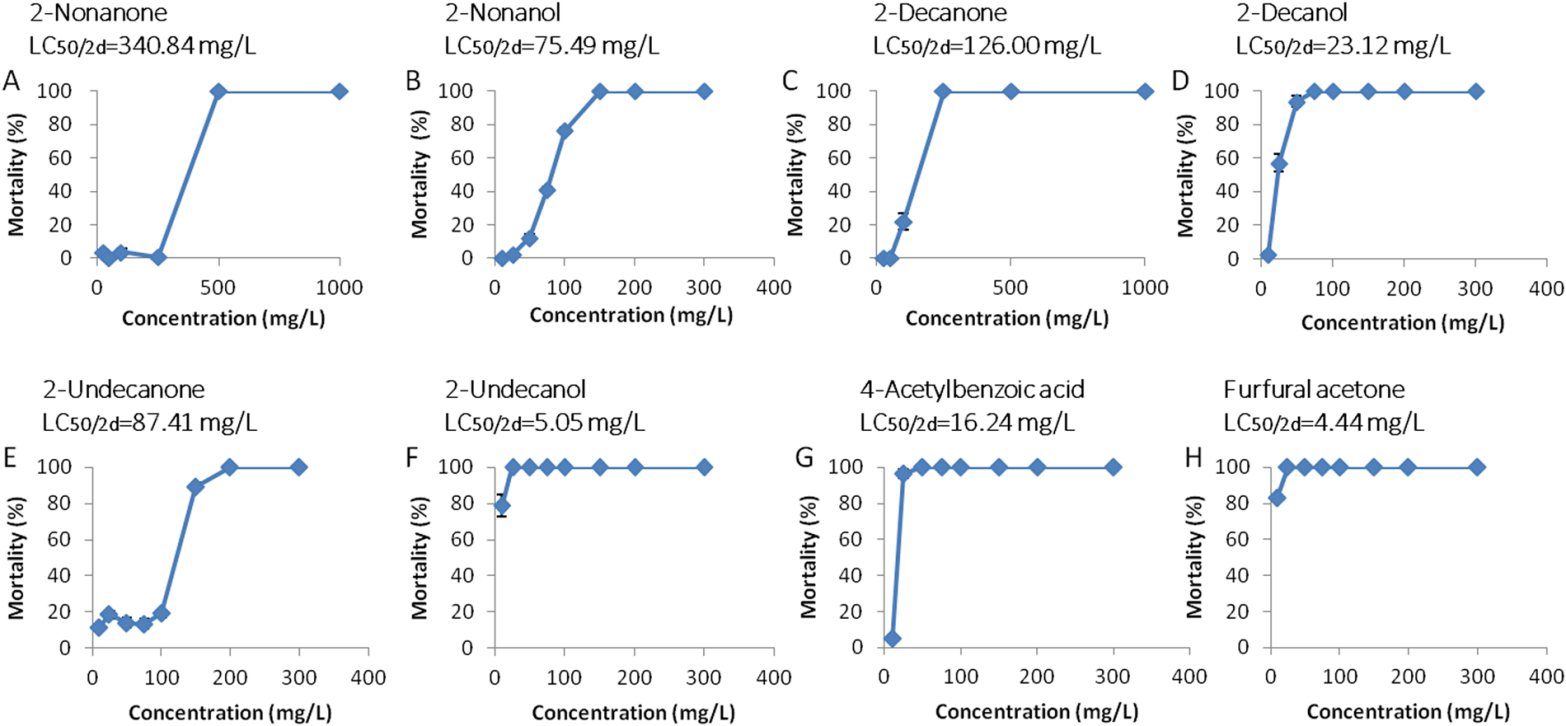
Curves of contact nematicidal activity against J2 juveniles of *M*. *incognita* immersed in solutions of (**A**) 2-nonanone, (**B**) 2-nonanol, (**C**) 2-decanone, (**D**) 2-decanol, (**E**) 2-undecanone, (**F**) 2-undecanol, (**G**) 4-acetylbenzoic acid, and (**H**) furfural acetone for 2 days. Each point represents the average percent number of dead J2 juveniles from 3 replications per treatment after elimination of natural death observed in the control. The data are shown as the mean ± SD (n = 3).

To confirm the hypothesis that the nematicidal VOCs are the primary nematicidal factor of KM2501-1, SPME-GC-MS was conducted to identify the VOCs produced by *P*. *polymyxa* KM2501-1. Apart from the 3 peaks produced by the KMB medium (Fig. 2A), 11 peaks were present in the chromatograms of the fermentation broth of KM2501-1 (Fig. 2B). Most of these were identified as alkanols, alkanones and acids. Of these, the ten that were commercially available were purchased for bioassays and listed in Table 2. Then J2 juveniles were used to test contact nematicidal activity against *M*. *incognita* immersed in treatment wells with VOCs at various concentrations. Furfural acetone, 2-undecanol, 4-acetylbenzoic, and 2-decanol acid were the most active, with LC_50/2d_ (50% lethal concentration at 2 days) values of 4.44, 5.05, 16.24, and 23.12 mg/L, respectively, followed by 2-nonanol, 2-undecanone, 2-decanone, and 2-nonanone, with LC_50/2d_ values of 75.49, 87.41, 126.00, and 340.84 mg/L, respectively (Fig. 3). The mortality rates of acetone and 2-heptanone against *M*. *incognita* were below 10% even at a concentration of 1,000 mg/L (data not shown).

**Table 2:** The information of authentic compounds used for bioassays.

Compounds	Chemical Abstracts Service Number	Manufacturer (Country)	Purity
Acetone	67-64-1	Sinopharm (China)	>99%
2-Heptanone	110-43-0	Tokyo Chemical Industry (Japan)	>98%
2-Nonanone	821-55-6	Tokyo Chemical Industry (Japan)	>98%
2-Nonanol	628-99-9	Tokyo Chemical Industry (Japan)	>98%
2-Decanone	693-54-9	Tokyo Chemical Industry (Japan)	>99%
2-Decanol	1120-06-5	Tokyo Chemical Industry (Japan)	>98%
4-Acetylbenzoic acid	586-89-0	Tokyo Chemical Industry (Japan)	>98%
Furfural acetone	623-15-4	Tokyo Chemical Industry (Japan)	>98%
2-Undecanone	112-12-9	Alladin (China)	>99%
2-Undecanol	1653-30-1	Tokyo Chemical Industry (Japan)	>98%

### Fumigant activity of VOCs against *M*. *incognita*

**Figure 4:**
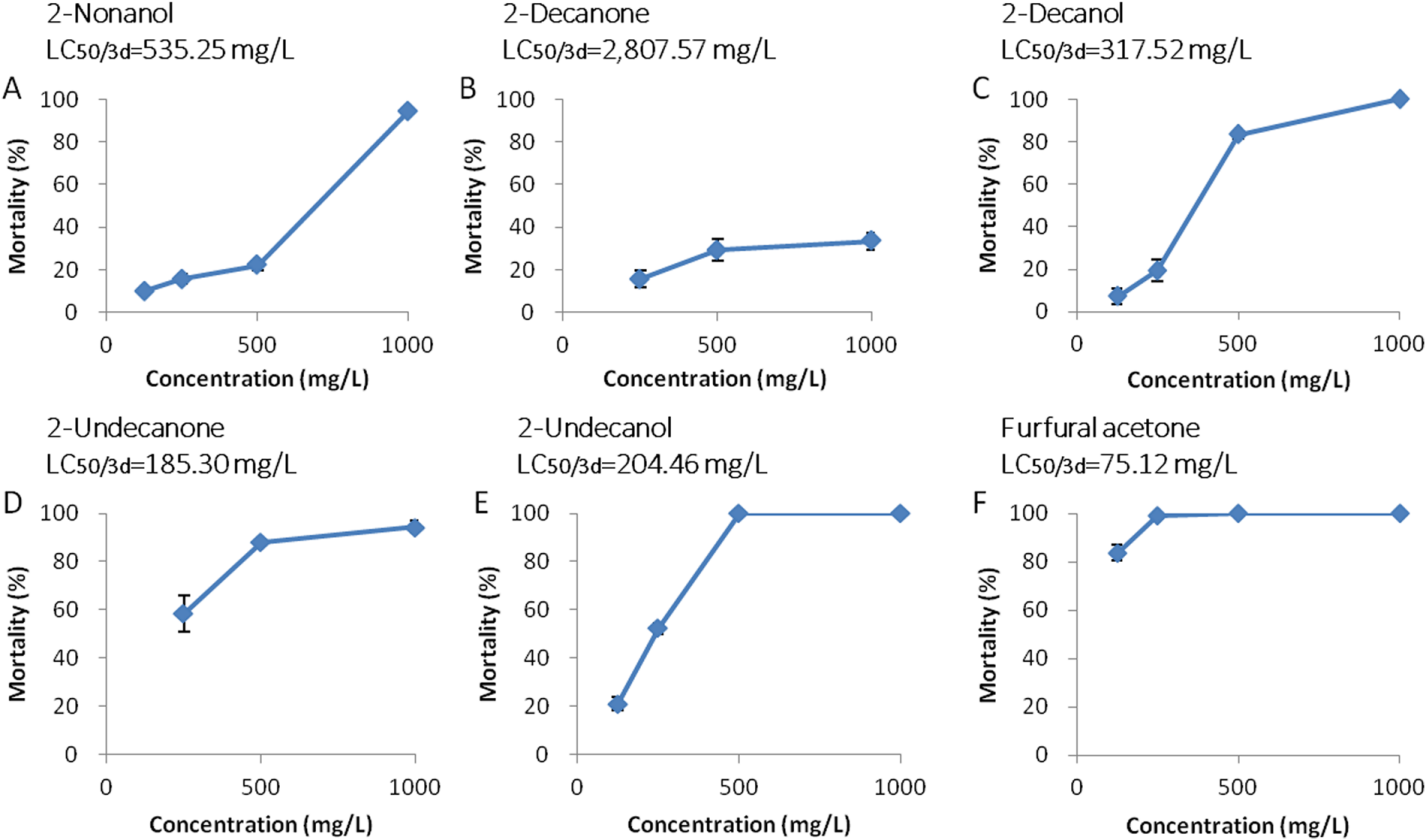
Curves of fumigant activity against J2 juveniles of *M*. *incognita* of (**A**) 2-nonanol, (**B**) 2-decanone, (**C**) 2-decanol, (**D**) 2-undecanone, (**E**) 2-undecanol, and (**F**) furfural acetone for 3 days. Each point represents the average percent number of dead J2 juveniles of 4 replications per treatment after elimination of natural death observed in the control. The data are shown as the mean ± SD (n = 4).

2-Nonanol, 2-decanone, 2-decanol, 2-undecanone, 2-undecanol, and furfural acetone also had fumigant activity against *M*. *incognita*. The most active VOC was furfural acetone, with an LC_50/3d_ of 75.12 mg/L, followed by 2-undecanone, 2-undecanol, 2-decanol, 2-nonanol and 2-decanone, with LC_50/3d_ values of 185.30, 204.46, 317.52, 535.25 and 2,807.57 mg/L, respectively (Fig. 4), furfural acetone was also the most active in the contact assay against *M*. *incognita* immersed in treatment wells. This is the first report that these 6 VOCs have fumigant activity against *M*. *incognita*.

### Chemotaxis of *M*. *incognita* towards VOCs

Using a population chemotaxis assay, we screened J2 juveniles of *M*. *incognita* for responses to the 10 VOCs listed in Table 2 at concentrations ranging from 1 to 10,000 mg/L. If the chemotaxis indexes (C.I.) of the VOCs at 5 concentrations are all not significantly different compared with the C.I. of control group (0 mg/L), it means that the activity of VOCs influencing the chemotaxis of J2 juveniles is variable. The results (Table 3) showed that acetone, 2-decanol, and furfural acetone acted as attractants to *M*. *incognita*, whereas 2-undecanone acted as a repellent. 4-Acetylbenzoic acid showed as an “attractant at low concentration and as a repellent at high concentration” (ALRH) towards to *M*. *incognita*. There were significantly difference among the C.I. values of some concentrations of acetone, 2-decanol, 4-acetylbenzoic acid, 2-undecanone, and furfural acetone (Table 3). Whereas, the C.I. of 2-heptanone, 2-nonanol, 2-nonanone, 2-decanone and 2-undecanol at all 5 concentrations are not significantly different compared to the C.I. of control group, so chemotaxis of *M*. *incognita* towards to 2-heptanone, 2-nonanol, 2-nonanone, 2-decanone and 2-undecanol was variable and the results are not shown.

**Table 3:** *Meloidogyne incognita* chemotactic response to volatile organic compounds (VOCs).

Concentrations (mg/L)	Chemotaxis index				
	Acetone	2-Decanol	Furfural acetone	2-Undecanone	4-Acetylbenzoic acid
0	0.014 ± 0.029 c	0.014 ± 0.029 b	0.014 ± 0.029 c	0.014 ± 0.029 a	0.014 ± 0.029 bc
1	0.104 ± 0.032 bc	0.057 ± 0.066 ab	0.289 ± 0.056 b	−0.372 ± 0.066 c	0.239 ± 0.050 a
10	0.153 ± 0.028 b	0.110 ± 0.053 ab	0.471 ± 0.036 a	−0.435 ± 0.066 c	0.099 ± 0.045 b
100	0.231 ± 0.033 ab	0.138 ± 0.008 a	0.282 ± 0.055 b	−0.142 ± 0.019 b	−0.062 ± 0.058 c
1000	0.266 ± 0.041 a	0.166 ± 0.019 a	0.376 ± 0.052 ab	−0.086 ± 0.037 ab	−0.222 ± 0.037 d
10000	0.169 ± 0.033 b	ND	0.444 ± 0.036 a	ND	−0.229 ± 0.020 d

ND, not detectable. The data are shown as the mean ± SD (n = 6). Duncan’s multiple range test was employed to test for significant differences between treatments at P < 0.05. Different lowercase letters indicate significant difference between treatments (P* < *0.05).

Table 3 shows data of concentrations up to only 1,000 mg/L for 2-decanol and 2-undecanone, whereas the other VOCs were tested at up to 10,000 mg/L. This is because at a concentration of 10,000 mg/L, *M*. *incognita* paralyzed in the buffer area of 2-undecanone (Fig. S2), whereas they were paralyzed in the test area and alive in the control area of 2-decanol (Fig. S3). The C.I. of these 2 groups at a concentration of 10,000 mg/L are not shown. However, the observation that VOCs of the 2-decanol and 2-undecanone groups killed *M*. *incognita* at 10,000 mg/L also is consistent with their fumigant activity.

### Comprehensive strategy of VOCs against *M*. *incognita*

**Figure 5:**
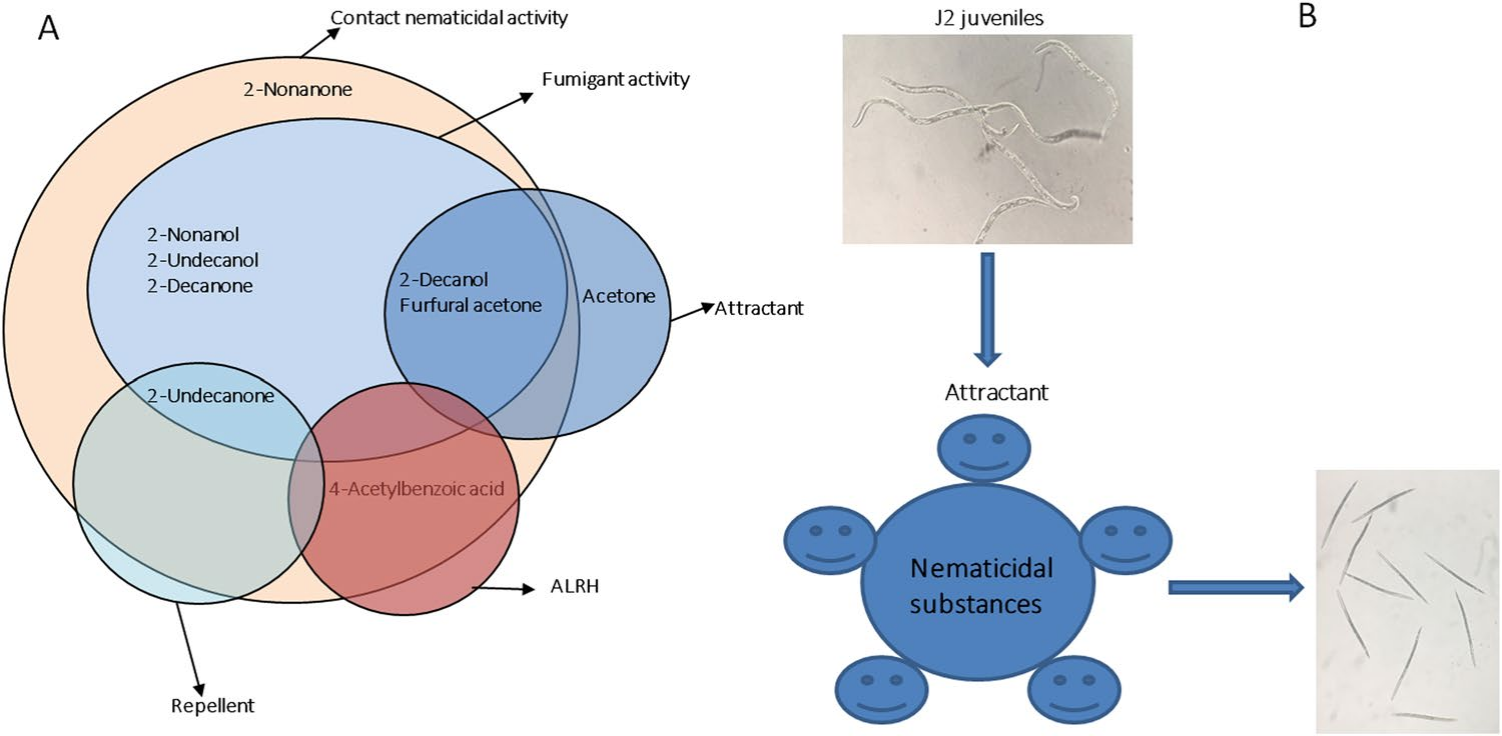
Complex strategies effective against PPNs: (**A**) “honey-trap” mode, fumigant mode and repellent mode and (**B**) Schematic diagram of “honey-trap” mode.

VOCs produced by KM2501-1 have comprehensive array of activities including contact nematicidal activity, fumigant activity, and activity affect the chemotaxis of the nematodes (Fig. 5A). As shown in Table 4, it is clear that VOCs like furfural acetone and 2-decanol have both contact nematicidal activity and fumigant activity against *M*. *incognita*, and also function as stable attractants. These VOCs have a novel “honey-trap” mode of action (Fig. 5B) in that they can attract *M*. *incognita* and then kill it by contact or fumigation. In contrast, VOCs like 2-undecanone had nematicidal and fumigant activity against *M*. *incognita*, but acted as stable repellent for *M*. *incognita* and could be applied to seeds or the roots of vegetables to initially repel, and subsequently kill invading nematodes. VOCs like 2-undecanol possessing both contact nematicidal and fumigant activity against *M*. *incognita* acted as fumigant mode, and could be more efficient when applied for nematode suppression for it could reach to the target nematodes that reside far from fumigants in the soil. VOCs like acetone, which do not themselves have nematicidal or fumigant activity but function as attractants, could be applied in combination with chemicals like abamectin to improve their efficiency.

**Table 4:** Behavior of *Meloydogyne incognita* towards volatile organic compounds (VOCs).

Compounds	Contact nematicidal activity	Fumigant activity against *M*. *incognita*	*M*. *incognita* chemotactic response
Acetone	−	−	attractant
2-Heptanone	−	−	variable
2-Nonanone	+	−	variable
2-Nonanol	+	+	variable
2-Decanone	+	+	variable
2-Decanol	+	+	attractant
2-Undecanone	+	+	repellent
2-Undecanol	+	+	variable
4-Acetylbenzoic acid	+	−	ALRH
Furfural acetone	+	+	attractant

+, has activity against *M*. *incognita*; −, no activity against *M*. *incognita*; ALRH, act as an attractant at low concentration and as a repellent at high concentration; variable, C.I. of the VOCs at all 5 concentrations are not significantly different compared with the chemotaxis index (C.I.) of control group.

### Nematicidal mechanism of VOCs and culture filtrate of KM2501-1 against *M*. *incognita*

**Figure 6:**
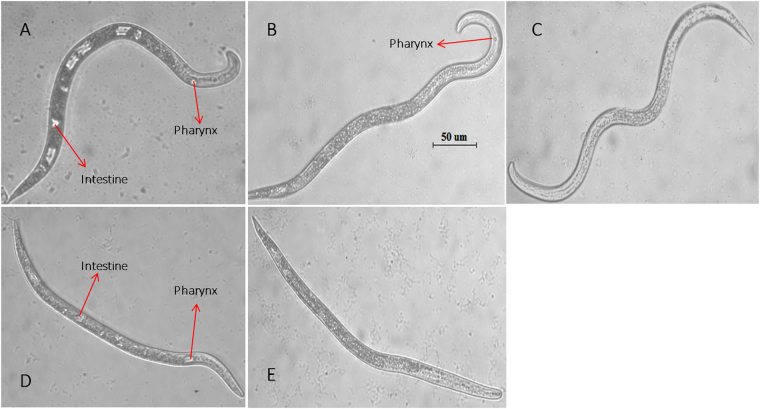
Morphological changes of the intestinal and pharyngeal tissues of living *M*. *incognita* treated with (**A**) solvent, (**B**) 250 mg/L 2-nonanone, (**C**) 100 mg/L 2-decanone, (**D**) KMB, and (**E**) culture filtrate of KM2501-1 for 48 h. All nematodes are shown at the same magnification (scan bar 50 μm).

VOCs kill nematodes by mechanisms that can affect the nervous system^28^, surface coat, intestine^8^, pharynx, or other tissues. Vacuoles were observed in the intestine of *M*. *incognita* after exposure to some VOCs (Fig. S4) and further investigated due to their resemblance to the effects of VOCs on other nematode tissues. We first assessed the pathological characteristics of *M*. *incognita* disposed by 2-nonanone and 2-decanone. J2 juveniles of *M*. *incognita* (50–60 per well) were exposed to 250 mg/L 2-nonanone or 100 mg/L 2-decanone for 48 h and then compared morphologically to control nematodes by optical microscopy (Fig. 6). We excluded dead nematodes from these observations because we wanted to avoid morphological changes that may have occurred as part of the death process. The results showed that the pharyngeal tissues of J2 juveniles had shrunken or even disappeared, and that the intestinal tissues became indistinct after treatment with 2-nonanone or 2-decanone. No disruption was observed in the intestine or pharyngeal tissues of the control group exposed only to solvent (distilled water). These results demonstrate clearly that 2-nonanone and 2-decanone disrupt the intestine and pharynx of *M*. *incognita*. Culture filtrate of KM2501-1 similarly disrupted the intestine and pharynx of *M*. *incognita* (Fig. 6), providing evidence that these VOCs are the primary nematicidal factor in strain KM2501-1.

## Discussion

*M*. *incognita* is an important plant pathogen causing severe damage to crops worldwide. Chemical nematicides are the primary means of control for plant parasitic nematodes, but their potential negative impacts on human health and the environment have led to a total ban or greatly restricted the use of such compounds. Researchers have therefore searched for decades for antagonistic microorganisms as an alternative to nematicidal chemicals, and many such fungi and bacteria have been reported^30,31^. However, the development of biological nematicides has been constrained by difficulties in commercial production and formulation^32^. Some nematicidal fungi are difficult to produce or are inhibited by soil^33^, and obligate bacteria like *Pasteuria penetrans* cannot easily be cultured^34^. Therefore, environmentally friendly, effective and affordable alternatives for PPN control remain urgently needed. The results of the present study indicate that *P*. *polymyxa* strain KM2501-1 has strong nematicidal activity against *M*. *incognita* and great potential for further development because as a sporeformer, it can easily be formulated for agricultural use.

In this study, 11 VOCs were isolated from strain KM2501-1, of which acetone and 2-heptanone had no contact nematicidal activity against *M*. *incognita*. These results are in line with an earlier report that 2-heptanone is inactive against *M*. *incognita* ^35^, but differ from a previous study indicating that acetone is active against *Panagrellus redivivus* and *Bursaphelenchus xylophilus* ^21^. When the nematicidal VOCs were tested individually over a range from 10 to 1,000 mg/L in the contact nematicidal experiment, clear dose response relationships were observed against *M*. *incognita*. These results are the first to show contact nematicidal activity of furfural acetone, 2-undecanol, 2-decanol, and 4-acetylbenzoic acid against *M*. *incognita*. Interestingly, there was a significant difference in the contact nematicidal activities of 2- alkanone and 2- alkanol homologues of different carbon chain length (Fig. 3). The LC_50/2d_ values of 2-nonanone, 2-decanone, and 2-undecanone were 340.84, 126.00, and 87.41 mg/L, respectively, while the LC_50/2d_ values of 2-nonanol, 2-decanol, and 2-undecanol were 75.49, 23.12, and 5.05 mg/L, respectively. When the carbon chain length was 9 to 11, the longer chain lengths of 2- alkanone or 2-alkanol had better nematicidal activity, similar to what was reported previously for nematicidal activity of 2-(1-alkyloxy)-1-ethanol homologues^36^ and alcohol homologues^37^. In addition, the 2-alkanols were more effective in nematode control than 2- alkanones of carbon length 9 to 11.

In contrast to the results of the contact nematicidal activity assay against *M*. *incognita*, some VOCs lacked fumigant activity even after 3 days. For example, 2-nonanone and 4-acetylbenzoic acid were active against *M*. *incognita* immersed in treatment wells, but showed no fumigant activity, with mortality below 20% even at a concentration of 1,000 mg/L after 3 days (data not shown). Fumigant activity differs from contact nematicidal activity against *M*. *incognita* immersed in treatment wells, and is of great importance for nematode control because it helps to reach target nematodes that reside far from nematicides in the soil^22^. The VOCs with both fumigant activity and contact nematicidal activity against *M*. *incognita* may be more efficient when applied for nematode suppression.

It has been reported that many VOCs have nematicidal^35,38^ and fumigant activity^39^, and affect the chemotaxis of the nematodes^40,41^, but VOCs with the comprehensive array of activities of those produced by KM2501-1 have not been previously described. The collective activities of these VOCs make KM2501-1 likely to be more effective as a nematicide. Especially effective was furfural acetone, with strong contact nematicidal (LC_50/2d_ = 4.44 mg/L) and fumigant activity (LC_50/3d_ = 75.12 mg/L), and the highest chemotaxis index among those tested (C.I. of 0.28 to 0.47). 2-Nonanone, 2-decanone, and the culture filtrate of KM2501-1 destroyed the integrity of the nematode pharynx and intestine, a mechanism that deserves to be explored further.

There also have been some reports about new modes of nematode control like the “Trojan horse” mechanism^42^ and the use of urea released by bacterial to mobilize nematode-trapping fungi to kill nematodes^43^. However, these mechanisms have only been tested with the model nematode *Caenorhabditis elegans*, unlike the “honey-trap” and repellent modes of action we presented here that were tested directly against *M*. *incognita*. Our results suggest that the “honey-trap” and repellent modes could be potentially used for PPN control.

In conclusion, *P*. *polymyxa* strain KM2501-1 provided good biocontrol of *M*. *incognita* due to production of a comprehensive array of VOCs with nematicidal, fumigant, and chemotactic activity. As a sporeformer, the strain itself could readily be formulated for agricultural application, while the VOCs it produces also have potential for development as pesticides, either alone or in combination to improve the performance of existing chemicals. The results of this study demonstrate that the VOCs produced by strain KM2501-1 exhibit a complex array of strategies against nematodes including a “honey-trap” mode, a fumigant mode and a repellent mode. For the “honey-trap” mode, furfural acetone lures *M*. *incognita* and then kills it. This “honey-trap” pattern of VOC–nematode interaction extends our understanding of the mechanisms of action of nematicidal VOCs. How the different mechanisms play roles in the various stages of host crop growth and interactions with nematodes should be subjects of further research.

## Materials and Methods

### Bacteria and nematodes

*Paenibacillus polymyxa* KM2501-1 was isolated from rhizosphere soil of buttercup (*Ranunculus*) polluted with recalcitrant organic compounds in Hukou county, Jiangxi province, China, and stored at −80 °C in our laboratory. The method that strain KM2501-1 isolated from rhizosphere soil of buttercup referred to the literature^44^ pubilished by our group. Strain KM2501-1 proved to be *Paenibacillus polymyxa* by sequencing its 16 S rDNA and constructing Phylogenetic trees of strain KM2501-1 and other 13 *Paenibacillus* or *Bacillus* strain based on 16 S rDNA (Fig. S5). The strain was grown on Kings medium B (KMB) agar plate at 28 °C for 48 h. Individual isolates were then inoculated into 100 mL KMB broth and incubated on a rotary shaker (180 rpm) at 28 °C in the dark for 48 h. Cultures were centrifuged and the supernatant solution was passed through a 0.22 μm nitrocellulose filter to prepare sterile culture filtrates (CF) for the assays described below. The bacterial of KM2501-1 was washed by sterile water for 3 times and then suspended in sterile water. The bacterial suspension (BS) in water (OD_600_ of undiluted bacterial suspension was 0.8) was prepared for the pot assays.

*M*. *incognita* was maintained on the roots of tomato (*Solanum lycopersicum*). Nematode eggs were isolated from the galls formed on infected tomato roots. To assess the nematicidal activity of KM2501-1 and VOCs, egg masses were peeled off from the root with needles and placed in water at 20 °C. Freshly hatched J2 juveniles were collected in sterile tube 3 days later and used in all of the assays.

### Activity *in vitro* of culture filtrates of KM2501-1 against *M*. *incognita*

To examine nematicidal activity, 120 μL of undiluted culture filtrate, dilutions of 1:1 (v/v, 1/2 × CF), or dilutions of up to 1:3 (v/v, 1/4 × CF) were transferred to 96-well plates and each well was filled with a freshly hatched suspension of approximately 30 J2 juveniles. KMB broth was used as a control (CK). Each treatment was replicated three times. Plates were covered with plastic lids, maintained in the dark at 20 °C, and dead *M*. *incognita* were counted after exposure under an inverted microscope. *M*. *incognita* was considered dead when no movement was observed for 2 s after contact with a needle. The percentages of dead nematodes observed were corrected by eliminating natural death in a negative control according to the Schneider-Orelli formula^45^.

### Nematicidal activity of *P*. *polymyxa* strains KM2501-1 VOCs

Nematicidal activity of *P*. *polymyxa* strain KM2501-1 VOCs was assayed in three-compartment Petri dishes according to the method of Gu *et al*.^21^ with some modifications. Briefly, the bacteria were cultured in KMB agar for 24 h at 28 °C in one compartment and a layer of 2% water agar (WA) was added to the other two compartments. After 24 h, about 200 nematodes of *M*. *incognita* were added to the two compartments with WA. Plates were immediately covered with lids to prevent the escape of the volatiles. After incubation at 25 °C in the dark for 24 h, the numbers of mobile and immobile nematodes recorded by counting under a microscope, and the total number of nematodes must be more than 100 nematodes per compartment. Immobile nematodes were immediately transferred to tap water to determine their potential for revival. As a control, KMB agar without KM2501-1 was added to one compartment of plates. The test repeated 4 times.

### Control efficiency of *P*. *polymyxa* strains KM2501-1 against *M*. *incognita* in the greenhouse

Plastic round pots (18 cm × 18 cm × 12.5 cm) were filled with about 1 kg sterile soil mixture (sand, field soil and organic matter, 1:1:1). One four-leaf stage tomato seedling was transplanted into each pot and incubated in the greenhouse at 22–25 °C. Each tomato seedling was irrigated around the roots with 10 mL of either *P*. *polymyxa* KM2501-1 culture filtrates (CF), a washed bacterial suspension (BS) in water (OD_600_ of undiluted bacterial suspension was 0.8), or just water (negative control) around the roots 2 days after transplanted. Two days later, about 2,000 J2 juveniles of *M*. *incognita* were inoculated into the rhizosphere soil of each seedling. The culture filtrate (CF) and bacteria suspension (BS) of KM2501-1 were each tested at 4 concentrations, with five seedlings at each concentration and control group (CK). Two replicates were set up for each treatment. Post-transplantation for 60 d, the severity of root galling was assessed^46^.

### Extraction of VOCs from fermentation broth of strain KM2501-1 by solid phase microextraction (SPME)

A new 75 mm CAR/PDMS SPME fiber (Supelco, Bellefonte, PA, USA) was conditioned with helium at 270 °C for 2 h prior to use. After each extraction cycle, the fiber was returned to the SPME needle to prevent contamination and conditioned again with helium at 270 °C for 20 min. Extractions were performed in 15 mL Supelco SPME vials filled with 9 mL bacterial culture containing a stir bar. The vials were clamped inside a thermostatic water bath placed on a hot stirrer. The SPME needle was allowed to pierce the septum and the fiber was exposed to the headspace of the vial for 90 min at 60 °C with constant magnetic stirring. The VOCs from 9 mL KMB broth were used as controls.

### Identification of nematicidal VOCs by gas chromatography-mass spectrometry (GC-MS)

A Hewlett Packard 7890GC/5975MSD (Agilent Technologies, USA) equipped with a HP-5MS capillary column was used to separate and identify the VOCs. The carrier gas was helium with a flow rate of 1 mL/min in split-splitless mode. The SPME fiber was inserted directly into the front inlet of the gas chromatograph and desorbed at 270 °C for 2 min. The oven temperature was programmed as follows: 40 °C for 2 min, 40–180 °C at a rate of 4 °C/min, 180–250 °C at 5 °C/min, and held at 250 °C for 6 min. The temperature of the transfer line and ion trap were 150 and 250 °C, respectively. Identification of VOCs was based on a comparison of the mass spectrum of the substance with standards in the GC/MS system data bank NIST08.L (National Institute of Standards and Technology). The experiment was conducted three times.

### Contact nematicidal activity of VOCs against *M*. *incognita*

Pure compounds of VOCs identified by GC-MS, namely 2-nonanol, 2-decanol, 2-undecanone, 2-undecanol, 4-acetylbenzoic acid, and furfural acetone, were individually subjected to dose response experiments against J2 juveniles over a range of 10–300 mg/L. Being the most inactive of the pure substances tested, acetone, 2-heptanone, 2-nonanone and 2-decanone were tested over a range of 25–1,000 mg/L. Pure compounds were prepared in ethanol and were successively diluted in distilled water containing the polysorbate surfactant Tween 20. Final concentrations of ethanol and Tween 20 in treatment wells never exceeded 1% and 0.1% (v/v), respectively.

To examine nematicidal activity *in vitro*, 120 μL of commercial VOCs at various concentrations were transferred to 96-well plates and then the wells were filled with J2 juveniles (approximately 30 *M*. *incognita*/well). Solvent carriers were used as controls. Each treatment was replicated three times. Plates were covered with plastic lids, maintained in the dark at 20 °C for 2 days, and dead *M*. *incognita* were counted after exposure under an inverted microscope. *M*. *incognita* was considered dead when no movement was observed for 2 s after touching with a needle. The percentages of dead nematodes observed were corrected by eliminating natural death in a negative control according to the Schneider-Orelli formula^45^.

### Fumigant activity of VOCs against *M*. *incognita*

The assay of fumigant activity was conducted by the method of Nikoletta^35^ with some modifications. A central well in each 96-well plate was filled with 200 μL test solution of a compound identified by GC-MS at a dose of 125–1,000 mg/L and 0 mg/L as negative control, and the four surrounding adjacent wells each received about 100 J2 juveniles suspended in 120 μL water. Mortality percentages in the four surrounding wells were recorded after 72 hours and the experiment was conducted four times. Immobile nematodes were immediately transferred to tap water to determine their potential for revival. The percentages of dead nematodes observed were corrected by eliminating natural death in a negative control according to the Schneider-Orelli formula^45^.

### Chemotaxis of the *M*. *incognita* towards commercial VOCs

**Figure 7:**
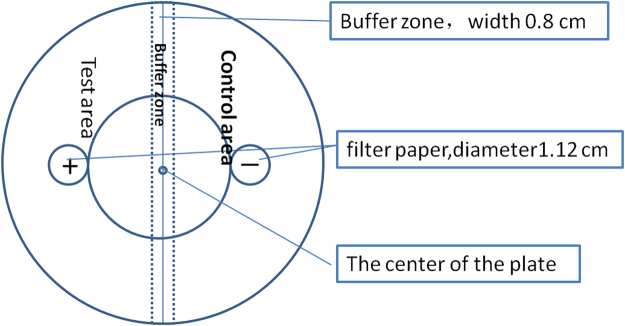
Schematic for chemotaxis assay plates A thin layer of agar in a 9-cm Petri dish was used as a substrate for chemotaxis. Roughly 200 J2 juveniles of *M*. *incognita* were placed near the center of the plate with a filter paper wetted with 30 μL VOCs at one side of the plate (test area in the figure) and a filter paper wetted with 30 μL solvent at the opposite side (control area in the figure). The distance between the center of filter paper and the midline of the plate was 25.6 mm. After 8 hours, the numbers of nematodes in the test and control areas were counted. Nematodes that remained within the 8-mm buffer zone at the midline were not counted for chemotaxis assays.

Chemotaxis was assessed on in 9-cm Petri dishes (Fig. 7) according to the method described by Bargmann *et al*.^41^ with some modifications. Ten mL of 2% water agar was poured into a 9-cm Petri dish with 3 areas: a buffer area including the 0.8 cm width of the middle line, a test area and a control area. Two 11.2 mm-diameter sterile filter paper discs were placed in the test and control areas respectively, with a distance between the center of the filter paper disc and the midline of the plate of 25.6 mm. Then 30 μL of different concentrations of VOCs were spotted onto the filter paper in the test area, and the same volume of solvent was spotted onto the filter paper in the control area. About 200–300 J2 juveniles of *M*. *incognita* (20 μL) were placed at the center of the plate. Chemotaxis assays were performed at 20 °C for 8 hours in the dark. The numbers of *M*. *incognita* in the test and control areas were then counted under an inverted microscope. Each VOC tested at 5 concentrations (1, 10, 100, 1000, and 10000 mg/L) and a control group (30 μL of 0 mg/L VOCs were spotted onto the filter paper in both test area and control area). The experiment was repeated 3 times, with 2 replicates at each concentration and control group at each test time.

For each VOC at each concentration, the chemotaxis index was equal to (the number of nematodes in the test area minus the number of nematodes in the control area) divided by (the number of nematodes in the test area plus the number of nematodes in the control area). For 0 < C.I. < 1, the VOC was considered as an attractant. For −1 < C.I. < 0, the VOC was considered as a repellent, and if the C.I. = 0, the VOC had no significant effect on chemotaxis.

### Microscopic observation

*M*. *incognita* was observed under an inverted microscope (Olympus, IX73) to determine the integrity of the intestine and the pharyngeal region. Under normal conditions, the pharynx and intestinal tissues were well-organized and could be seen clearly.

### Statistical analysis

Data were corrected by Schneider-Orelli formula^45^ and then analyzed using SPSS (Statistical Package for the Social Sciences), version 17.0 software (SPSS, Chicago, IL, USA). LC_50_ values were calculated using PROBIT analysis^47^ and the data shown as the mean ± Standard Deviation (SD) (n ≥ 3). Duncan’s multiple range test was employed to test for significant differences in pot experiment, chemotaxis experiment and experiment of nematicidal activity of *P*. *polymyxa* KM2501-1 culture filtrate between treatments at P < 0.05. Different lowercase letters indicate significant difference between treatments (P < 0.05). Statistical comparisons in other experiment between two values were performed with a *t*-test, and significant differences were determined according to a threshold of *P < 0.05; **P < 0.01; ***P < 0.001.

## Electronic supplementary material

Supplementary Information
