# Supplementary material for: Volatile organic compounds from Paenibacillus polymyxa KM2501-1 control Meloidogyne incognita by multiple strategies
Source: Sci Rep. 2017 Nov 24;7:16213. doi: 10.1038/s41598-017-16631-8 (PMC5701253; doi:10.1038/s41598-017-16631-8)

**Volatile organic compounds from** ***Paenibacillus polymyxa* KM2501-1 control *Meloidogyne incognita* by multiple strategies**

Wanli Cheng^1^, Jingyan Yang^1^, Qiyu Nie^1^, Dian Huang^1^, Chen Yu^1^, Longyu Zheng^1^, Minmin Cai^1^, Linda S. Thomashow^2^, David M. Weller^2^, Ziniu Yu^1^, Jibin Zhang^1^*

^1^State Key Laboratory of Agricultural Microbiology and National Engineering Research Center of Microbial Pesticides, College of Life Science and Technology, Huazhong

Agricultural University, Wuhan 430070, Hubei, China

^2^United States Department of Agriculture, Agricultural Research Service, Wheat Health, Genetics and Quality Research Unit, Pullman, WA 99164-6430, USA

*Correspondence:

Jibin Zhang, State Key Laboratory of Agricultural Microbiology, College of Life Science and Technology, Huazhong Agricultural University, Wuhan 430070, Hubei, China.

E-mail: zhangjb@mail.hzau.edu.cn

Fax: 86-27-87287254; Tel: 86-27-87287701

**Figure S1. The tomato roots of the (A) control (CK), (B) 1× culture filtrate of *P. polymyxa* KM2501-1 treated groups , and (C) 1× bacterial suspension of *P. polymyxa* KM2501-1 treated groups in the greenhouse experiment.**

**Figure S2. J2 juveniles of *M. incognita* paralyzed in the buffer area of the 10000 mg/L 2-undecanone treatment under the chemotaxis experiment.**

**Figure S3. J2 juveniles of *M. incognita* were (A) paralyzed in the test area, while (B) alive in the control area of the 10000 mg/L 2-decanol treatment under the chemotaxis experiment.**

**Figure S4. The pathological characteristics of *M. incognita* treated with (A) VOCs, or (B) solvent (CK).** **Figure S5. A phylogenetic tree based on 16S rDNA shows the inferred evolutionary relationship between the strain KM2501-1 and other *Panebacillus* and *Bacillus* species (sequences downloaded from http:// www.ncbi.nlm.nih.gov/).**

**Table S1. Nematicidal activity of *P. polymyxa* KM2501-1 culture filtrate (CF) at various concentrations and control group (CK) against *M. incognita* immersed in treatment wells.**

| Treatment | Mortality (%) | | |
| --- | --- | --- | --- |
|  | 24h | 48h | 72h |
| CK | 0±0 e | 4.67±4.51 e | 5.12±1.49 e |
| 1/4×CF | 33.33±2.89 d | 58.33±2.89 b | 80.00±5.00 a |
| 1/2×CF | 46.59±11.00 c | 80.35±3.52 a | 84.39±2.73 a |
| 1×CF | 66.24±5.19 b | 84.80±6.61 a | 87.66±8.72 a |

The data are shown as the mean ± SD (n = 3). Duncan’s multiple range test was employed to test for significant differences between treatments at P <0.05. Different lowercase letters indicate significant difference between treatments (P < 0.05).

**Figure S1**


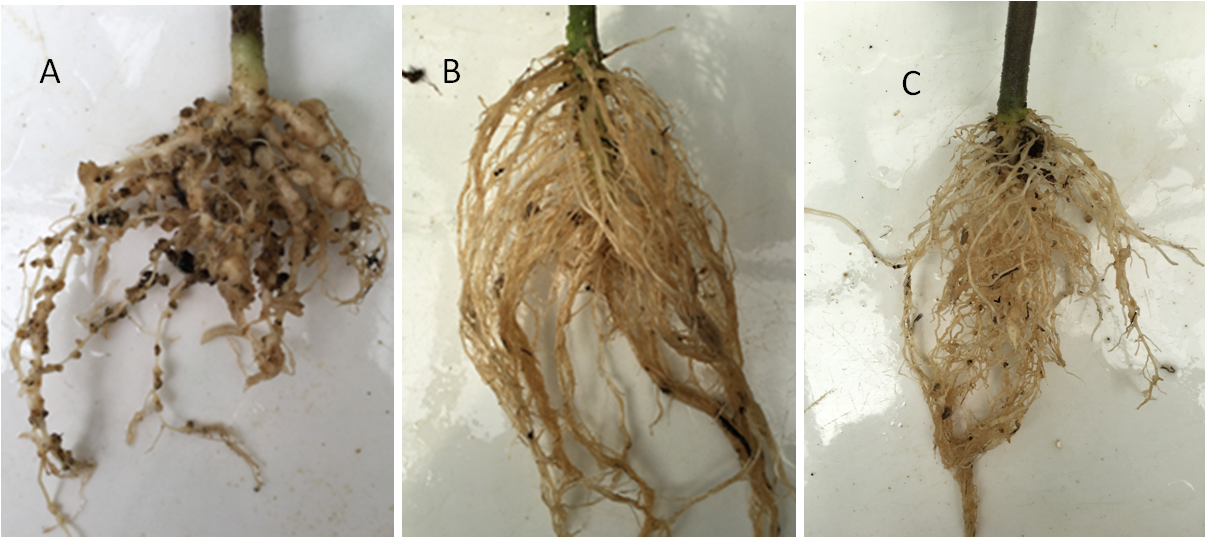


**Figure S2**

**
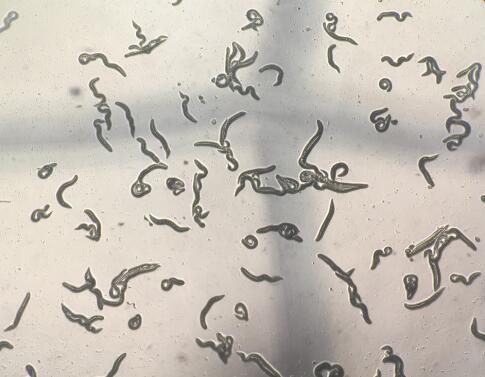
**

**Figure S3**

**
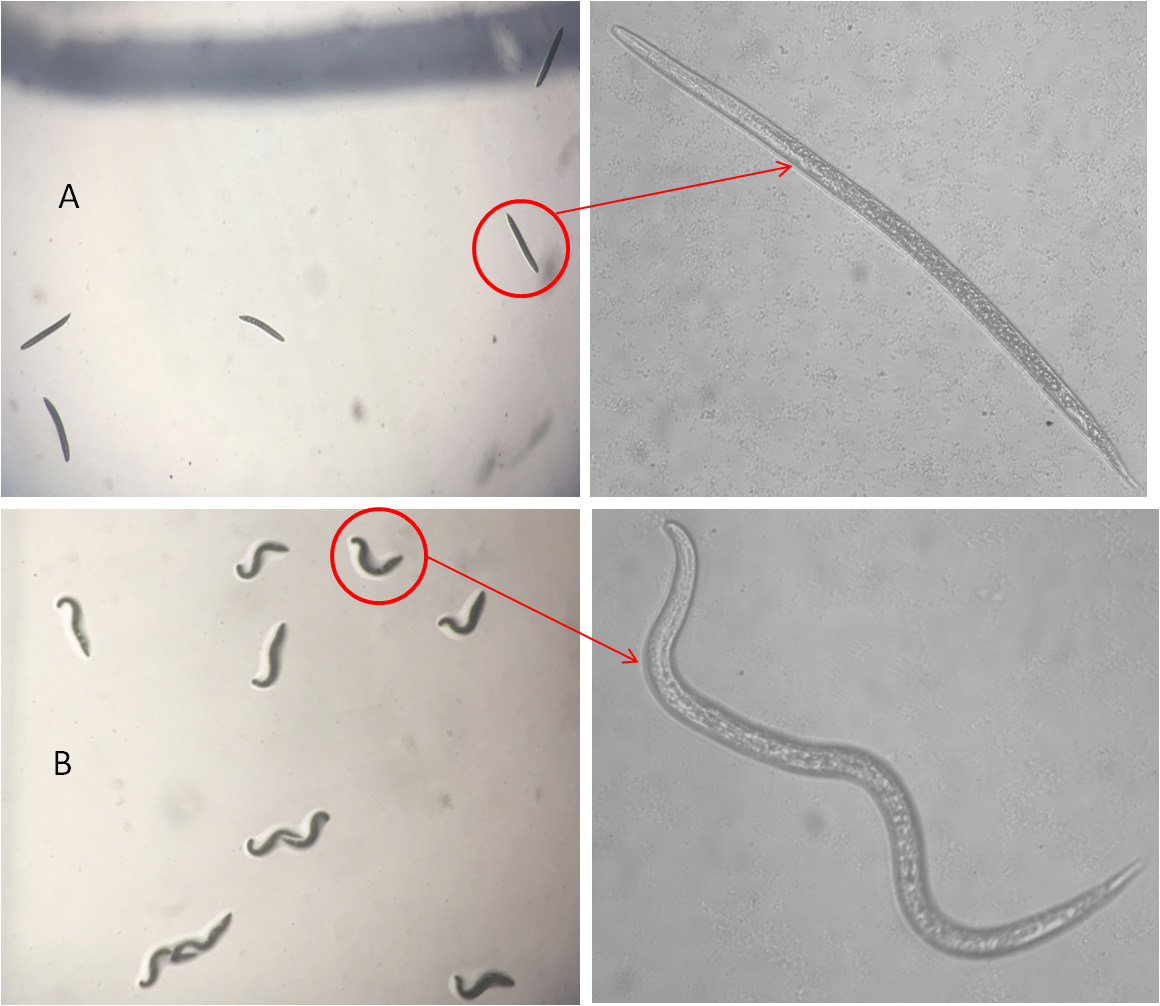
**

**Figure S4**

**Figure S5**


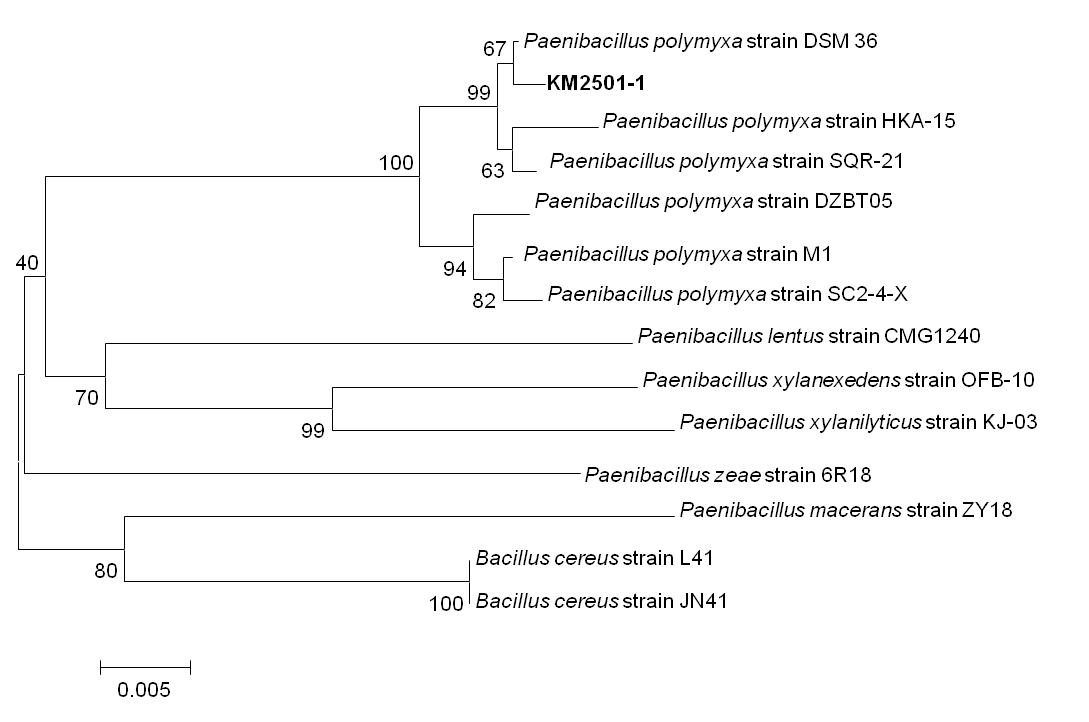

Supplement: Supplementary file 1 — Supplementary Information [file 41598_2017_16631_MOESM1_ESM.doc]
